# Engineering of Removing Sacrificial Materials in 3D-Printed Microfluidics

**DOI:** 10.3390/mi9070327

**Published:** 2018-06-28

**Authors:** Pengju Yin, Bo Hu, Langlang Yi, Chun Xiao, Xu Cao, Lei Zhao, Hongyan Shi

**Affiliations:** 1 School of Life Science and Technology, Xidian University, Xi’an 710126, China; pjyin@stu.xidian.edu.cn (P.Y.); llyi@stu.xidian.edu.cn (L.Y.); 1995xiaochun@gmail.com (C.X.); Xu.Cao@Dartmouth.edu (X.C.); 2 Library, Xidian University, Xi’an 710126, China

**Keywords:** 3D printing, sacrificial materials, microfluidics, removing efficiency, quantification

## Abstract

Three-dimensional (3D) printing will create a revolution in the field of microfluidics due to fabricating truly three-dimensional channels in a single step. During the 3D-printing process, sacrificial materials are usually needed to fulfill channels inside and support the printed chip outside. Removing sacrificial materials after printing is obviously crucial for applying these 3D printed chips to microfluidics. However, there are few standard methods to address this issue. In this paper, engineering techniques of removing outer and inner sacrificial materials were studied. Meanwhile, quantification methods of removal efficiency for outer and inner sacrificial materials were proposed, respectively. For outer sacrificial materials, a hot bath in vegetable oil can remove 89.9% ± 0.1% of sacrificial materials, which is better than mechanics removal, hot oven heating, and an ethanol bath. For inner sacrificial materials, injecting 70 °C vegetable oil for 720 min is an optimized approach because of the uniformly high transmittance (93.8% ± 6.8%) and no obvious deformation. For the industrialization of microfluidics, the cost-effective removing time is around 10 min, which considers the balance between time cost and chip transmittance. The optimized approach and quantification methods presented in this paper show general engineering sacrificial materials removal techniques, which promote removing sacrificial materials from 3D-printed microfluidics chips and take 3D printing a step further in microfluidic applications.

## 1. Introduction

Over the past decade, the three-dimensional (3D) printing technique has emerged to improve the fabrication of microfluidic devices [1,2,3,4], which is believed to have created a revolution in microfluidics [5,6,7,8,9]. 3D printing, which does not depend on masks to create the microfluidic channels, takes inputs from computer-aided design (CAD) software, which is able to produce fully 3D objects [10,11,12,13]. The advantages of 3D-printed microfluidic chips, in contrast to soft lithography, are their low cost, high speed, and especially fully 3D channels [14,15,16,17]. Mostly, 3D printers fabricate microfluidics with three main methods: photopolymer inkjet printing [18,19,20,21], fused deposition modeling [22,23], or stereo lithography [24,25,26]. Compared to the other methods, photopolymer inkjet printing shows more benefits in the area of microfluidic chip fabrication due to its higher printing speed and precision [27,28]. Using a photopolymer inkjet printer to fabricate microfluidic chips, two types of sacrificial materials are added during the process. Outer sacrificial materials are added to support the structure of the chip outside and inner sacrificial materials are added to support the micro-channel inside. Although sacrificial materials have been widely used in 3D printing, easy and effective removal methods are not well-studied [29,30].

Removing sacrificial materials is crucial to the quality of microfluidic chips. Unfortunately, many companies and labs do not pay enough attention to developing standard methods for removing sacrificial materials and quantization methods for removing efficiency. Hongyi Yang et al. evaluated the performance of a Projet 3D printer with micro features, surface roughness, and printing accuracy [31]. In the removing steps for the sacrificial materials, the 3D-printed structure was just heated above the melting temperature to remove the sacrificial materials. This method is obviously convenient to remove the sacrificial material which supports the device outside. However, it may fail to remove most of the inner sacrificial material inside the micro-channels without injecting solvents (or removers) into the micro-channels. Sochol et al. presented how to remove outer and inner sacrificial materials in a centimeter-sized channel of a 3D-printed component [18]. They used dye-colored fluids to validate the channel, but there were no quantification methods to evaluate the removal efficiency.

In this paper, an engineering technique for removing outer and inner sacrificial materials is proposed. To evaluate the different removing approaches, quantification methods of removal efficiency for removing outer and inner sacrificial materials are presented. Based on these quantification methods, the approaches were optimized for removing outer and inner sacrificial materials. Finally, the engineering techniques were tested and verified in several 3D-printed microfluidic chips with different micro-channel cross-sections.

## 2. Materials and Methods

### 2.1. Chemicals and Materials

Ethanol (95%, analytical reagent) was purchased from Guangdong Guanghua Sci-Tech Co., Ltd. (Shantou, China) Vegetable oil was produced by the Xi’an Aiju Grain and oil industry group. Air was not processed before use. All water used in the experiment was deionized water.

### 2.2. Fabrication of 3D-Printed Microfluidic Chips

The first step of fabricating 3D-printed microfluidic chips is designing 3D chip models by a computer-aided design (CAD) software package. When designing the 3D model, the geometry of the microfluidic channel should be designed. Once the 3D model has been created, it needs to be converted to the standard 3D-printing file format called standard triangle language (STL). STL files describe the 3D model’s surface geometry by triangular sections, the number of which define the resolution of the 3D digital model. Before printing, the STL file needs to translate the model into a series of thin layers and produces a G-code file, which is the instruction file of a specific 3D printer.

In this paper, a serpentine microfluidic channel with an inlet and three outlets was used to validate the sacrificial materials removing efficiency. All microfluidic chips are designed and drawn by SolidWorks (SolidWorks 2014, Dassault Systèmes, Vélizy-Villacoublay, France) in STL format and printed via a Projet 3500 HD MAX (3D systems, Rock Hill, SC, USA) with Visjet^®^ M3 (3D systems) Crystal build materials and Visjet^®^ R S300 sacrificial materials. Another build material, VisiJet^®^ Techplast material, was used to test the removers of ethanol and oil. The resolution of each printed layer by the highest precision mode achieves 16 μm.

### 2.3. Four Methods to Remove Outer Sacrificial Materials

The microfluidic chips were treated by four methods to remove outer sacrificial materials. For each method, chips were weighed by an analytical balance before and after removal. The initial mass of the chips and the mass of the chips after removing were recorded, respectively. For mechanical removal, the knife tool was used to remove outer sacrificial materials as much as possible. For hot oven removing, chips were placed in a 70 °C oven for 10 min with glassware to melt the sacrificial materials. For the ethanol and oil baths, a hot plate was used to heat the ethanol and oil in a beaker. Chips were placed in the beaker for 10 min to remove the outer sacrificial materials. Each experiment was repeated three times.

### 2.4. Inner Sacrificial Materials Removal with Three Removers

A hot plate was used to heat the vegetable oil to 70 °C in a beaker. Chips were placed in the beaker to melt the sacrificial materials in the channel. A constant-flow pump (Shanghai QiTi analytic instrument Inc., Shanghai, China) was used to pump three removers (air, ethanol, and vegetable oil) to the inner chip channels for 10 min with the rate of 0.1–0.3 mL/min. Each experiment was repeated three times.

### 2.5. Inner Sacrificial Materials Removal under Different Temperatures

A hot plate was used to heat vegetable oil in a beaker to different temperatures (60 °C, 70 °C, and 80 °C). For each temperature, a constant-flow pump was used to pump vegetable oil to the inner chip channels for 10 min. After this, chip images were captured by a biological microscope. Each experiment was repeated three times.

### 2.6. Inner Sacrificial Materials Removal under Different Times

Three-dimensional (3D)-printed microfluidic chips were placed in a heated beaker (70 °C) and vegetable oil was pumped to the inner chip channel by a constant-flow pump for different times (1, 3, 10, 20, 40, 120, 360, and 720 min). For each time, the constant-flow pump was used to pump vegetable oil to the inner chip channels at 70 °C. After this, chip images were captured by a biological microscope. Each experiment was repeated three times.

### 2.7. Sacrificial Materials Removal from Microfluidic Chips with Different Cross-Sections

Microfluidic chips with different cross-sections (rectangle, circle, half-circle, and triangle) were designed and printed as described above. For each cross-section chip, images were captured by a biological microscope without removing the inner sacrificial materials. Those are the 0 min results. Then, a hot plate was used to heat the vegetable oil to 70 °C in a beaker. For 1 min results, different cross-section chips were placed in the beaker and vegetable oil was pumped to the inner chip channel by a constant-flow pump for 1 min. For 3 min results, other different cross-section chips were placed in the beaker and vegetable oil was pumped to the inner chip channel for 3 min. The same procedures were done for the experiments of 5, 10, and 20 min. Each experiment was repeated three times.

### 2.8. Gray Scale Value Extracting

A biological microscope and camera were used to capture chip images under the same setting. The images were used to calculate the gray scale value of the chips. For each single image, three areas (50 pixel × 50 pixel for each area) were chosen randomly in and out of the channel area. The gray value of each area was extracted for the transmittance calculation.

### 2.9. Data Analysis and Statistics

The removal efficiency of outer sacrificial materials and the transmittance of removing inner sacrificial materials were performed at least in triplicate. The primary data of these two quantification methods are presented as the means with standard deviations.

### 2.10. Characterization

For outer sacrificial materials, an analytical balance (BSA224S, Sartorius Inc., Shanghai, China) was used to weigh the chip before and after sacrificial materials removal. To capture chip images, a Bk 6000 biological microscope (Optec Instrument Co., Ltd., Chongqing, China) and a GS3-U3 camera (Point Grey Research Inc., Richmond, BC, Canada) were used.

## 3. Results

### 3.1. 3D-Printed Microfluidic Chips

**Figure 1:**
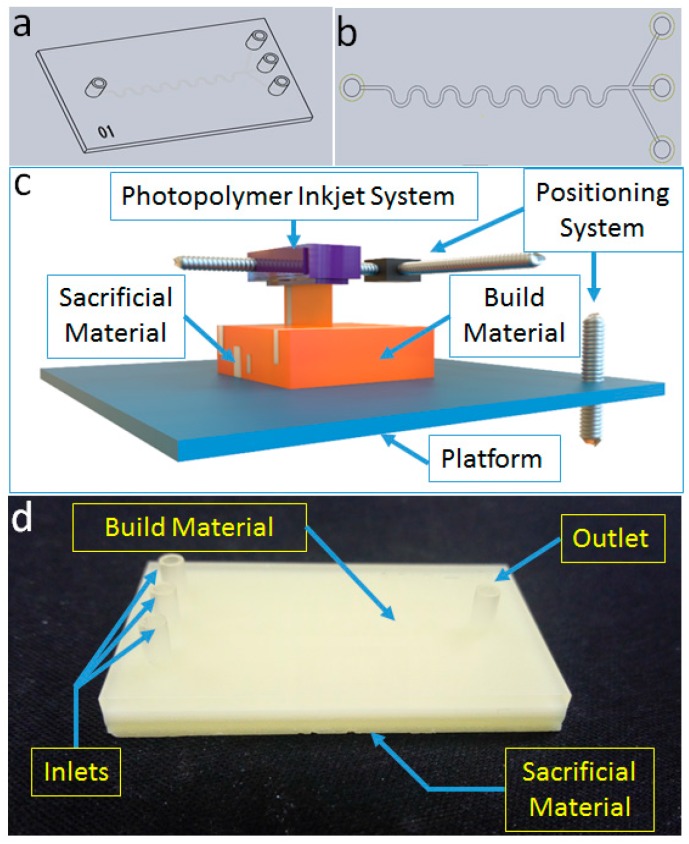
Design and fabrication of three-dimensional (3D)-printed microfluidic chips. (**a**) A microfluidic chip with a serpentine channel. (**b**) A 3D chip model designed by SolidWorks. (**c**) Schematic of the polyjet 3D printer with three main parts, including the photopolymer inkjet system, the positioning system, and the forming platform. (**d**) 3D printed chips with build materials and sacrificial materials.

In order to study the removal process of sacrificial materials, microfluidic chips with a serpentine channel were designed and drawn in SolidWorks. All 3D models of the microfluidic chips were printed by a photopolymer inkjet 3D printer under the highest precision mode. Serpentine channel microfluidic chips with one inlet and three outlets were designed (Figure 1a,b). The width and height of the channel were 400 μm and 200 μm, respectively. After conversion to STL format, 3D microfluidic models were inputted to the photopolymer inkjet 3D printer. The photopolymer inkjet 3D printer used in this study has three main parts, including a photopolymer inkjet system, a positioning system, and the forming platform (Figure 1c). Two kinds of materials, build materials and sacrificial materials, are normally used for printing. The solid parts of the 3D model are printed with build materials, while the inner channels and outer parts, which need to be supported, are printed with sacrificial materials. After printing, the outer and inner sacrificial materials should be removed to obtain the available microfluidic chips (Figure 1d).

### 3.2. Outer Sacrificial Materials Removing

**Figure 2:**
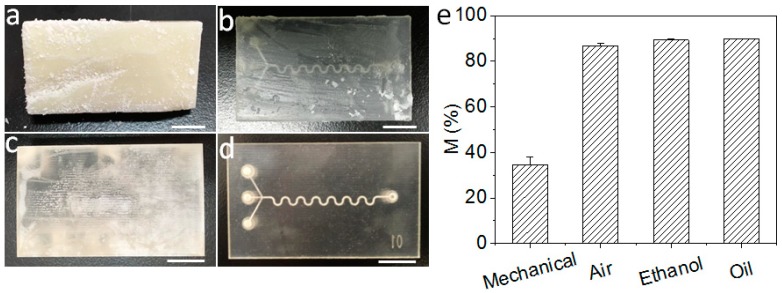
Four approaches to removing outer sacrificial materials. (**a**) Mechanical removing, (**b**) Removing outer sacrificial materials in a hot oven, (**c**) 95% hot ethanol, (**d**) hot vegetable oil, and (**e**) Mass reductions with the four approaches. The scale bar is 1 cm.

Four approaches, including mechanical removal, removal of sacrificial materials in a hot oven, a hot bath in 95% ethanol, and a hot bath in vegetable oil, were used to remove outer sacrificial materials. Mechanical removal is the simplest way to remove outer sacrificial materials without complex instruments. The results show that the mechanical approach cannot remove outer sacrificial materials efficiently due to the hardness at room temperature (Figure 2a). Therefore, to clear outer sacrificial materials, the chip needs to heat and melt the outer sacrificial materials first. Heating in a hot oven is used as another approach to remove outer sacrificial materials. A hot oven can remove most outer sacrificial materials, but a thin layer of sacrificial materials remains in the chip (Figure 2b). In consideration of the sacrificial materials dissolving in an organic solvent, a hot bath in an organic solution may be a better approach. Thus, 95% ethanol and vegetable oil were chosen to remove the outer sacrificial materials (Figure 2c,d). In order to valuate these four outer sacrificial materials removal approaches, a quantification method for removal efficiency is developed.

The outer sacrificial materials are placed around the chip to support the whole printed geometry. The weight of the sacrificial materials takes up a large proportion of the raw chip. In order to develop a quantification method to evaluate the removal efficiency of outer sacrificial materials, the mass reduction (*R*) of the weight before and after a removal approach is calculated as follows:

(1) $$R=\frac{M_{1}-M_{2}}{M_{1}-M_{0}}\times 100\%$$

*M*_0_ is the theoretical mass without outer sacrificial materials, which is calculated by the volume and density of the printed chip and inner sacrificial materials. *M*_1_ is the chip mass with inner and outer sacrificial materials, which is obtained before the removing process. *M*_2_ is the chip mass after removing the outer sacrificial materials with different approaches.

Based on this quantification method, the removal efficiencies of outer sacrificial materials of the four approaches are evaluated (Figure 2e). The chips’ mass reduction by mechanical removing is 34.4% ± 3.5%. It is shown that the greater parts of the outer sacrificial materials still remain. The chips’ mass reduction by hot oven is 86.8% ± 1.2%, which is higher than that of mechanical removal. Additionally, the chips’ mass reduction by a hot bath in ethanol and in vegetable oil are 89.3% ± 0.7% and 89.9% ± 0.1%, respectively.

Mechanical removal and removal in a hot oven are easy to perform and have a low mass reduction. Chip mass reduction by a hot bath in ethanol and a hot bath in vegetable oil are almost the same. It indicates that both hot ethanol and vegetable oil can remove outer sacrificial materials clearly. However, the pictures have shown that ethanol could blur the surface of the chip and oil makes the chip clear (Figure 2c). Therefore, hot vegetable oil can dissolve sacrificial materials without damaging the build materials. A hot bath in vegetable oil is the best way to remove outer sacrificial materials among the four methods present above (Figure 2d). The optimized method to remove outer sacrificial materials is a hot bath in vegetable oil.

### 3.3. Removing Inner Sacrificial Materials with Different Removers

**Figure 3:**
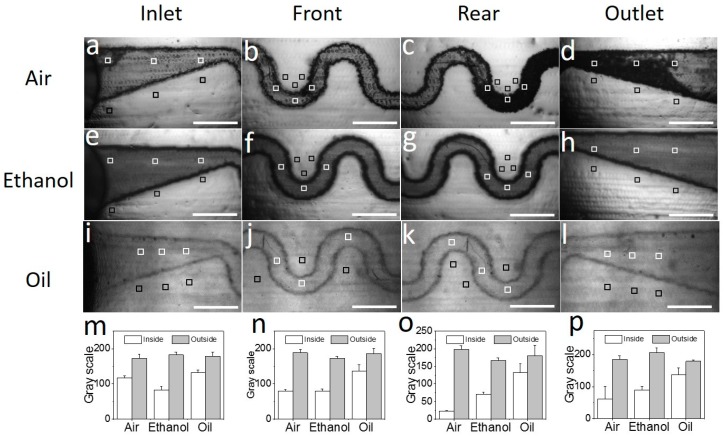
Inner sacrificial materials removal with three removers. (**a**–**d**) Inject air into the heating chip and take pictures at the (**a**) inlet, (**b**) front part, (**c**) rear part, and (**d**) outlet under the microscope. (**e**–**h**) Inject ethanol into a hot bath with the chip and take pictures at the (**e**) inlet, (**f**) front part, (**g**) rear part, and (**h**) outlet under the microscope. (**i**–**l**) Inject vegetable oil into a hot bath with the chip and take pictures at the (**i**) inlet, (**j**) front part, (**k**) rear part, and **(l**) outlet under the microscope. (**m**–**p**) Transmittance results at the (**m**) inlet, (**n**) front part, (**o**) rear part, and (**p**) outlet after processing by air, ethanol, and vegetable oil. The scale bar is 1 mm.

Removing inner sacrificial materials is harder than removing outer sacrificial materials due to the micro-sized channels in the microfluidic chips. To remove the sacrificial materials in the channel, different removers (air, 95% ethanol, and vegetable oil) were injected into the channel and the chip was heated to above the melting point of the sacrificial materials simultaneously. Pumping air into the heating chip, most melted materials can be pushed out of the channel (Figure 3a–d, Figure S1), but some materials remain in the rear part and outlet of the channel (Figure 3c,d, Figure S2). Injecting 95% ethanol into the heating chip, the melted sacrificial materials can be dissolved and removed out of the chip (Figure 3e–h, Figures S3 and S4). Meanwhile, the chip surface is also blurred by ethanol as observed above. Using vegetable oil as a remover, the sacrificial materials can be dissolved and removed out of the microchannel without chip damage (Figures S5 and S6).

In order to evaluate the inner sacrificial materials removal results quantitatively, a quantification method is necessary. As we know, the build materials of a 3D-printed chip are normally light permeable, and the sacrificial materials are light tight. Thus, microchannel transmittance is selected as a quantified parameter to evaluate the removal efficiency of inner sacrificial materials. For calculating the channel transmittance, microscopic images of microfluidic chips are acquired under the same conditions. Additionally, the gray scale values of six areas (Figure 3a–i), which are chosen equally and randomly inside and outside the channel area, are extracted from the images. The average gray values of these three areas are presented in Figure 3m–p. For every single microfluidic chip, the gray scale values of four parts are calculated: the inlet, front part of the chip, rear part of the chip, and the outlet. Then, the transmittance (*T*) of the channel can be calculated as follows:

(2) $$T=\frac{X-Z}{Y-Z}\times 100\%$$

where *X* is the gray scale value inside the channel area, *Y* is the gray scale value outside the channel area, both of the *X* and *Y* values are between 0–255 in an 8-bit image, and *Z* is the gray scale value without light transmission (i.e., 0).

The transmittances of the four parts are calculated by Equation (2). After processing by air, the transmittances of the four parts are 67.7% ± 2.9%, 42.8% ± 3.0%, 11.6% ± 0.4%, and 32.5% ± 19.3% (Figure S2), respectively. The transmittances of the inlet and front part are higher than those of the rear part and outlet. Injecting air into the hot chip channel can remove the sacrificial materials in the front part of the chip. However, there are still residual sacrificial materials in the rear part of the chip. After processing by ethanol, the transmittances of the four parts are 45.4% ± 3.6%, 46.6% ± 3.8%, 42.2% ± 2.7%, and 43.4% ± 2.0% (Figure S4), respectively. There is no obvious difference among the four parts. After processing by oil, the transmittances of the four chip parts are 73.7% ± 2.5%, 73.0% ± 4.6%, 73.06% ± 13.3%, and 76.1% ± 12.4% (Figure S5), respectively. There are no obvious differences among the four parts processed by ethanol.

**Figure 4:**
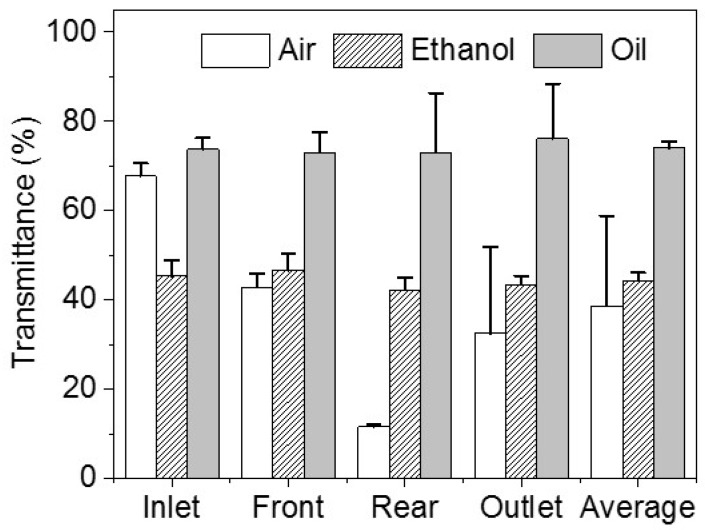
Transmittance of the chips after removing inner sacrificial materials with different removers. The transmittances of four parts are calculated: the inlet of the chips, the front part of the chips, the rear part of the chips, and the outlet of the chips. The average transmittance of the four parts represents the transmittance of the chips.

The average chip transmittances using these three removers are 38.7% ± 20.2%, 44.4% ± 1.7%, and 74.0% ± 1.3% (Figure 4), respectively. As described above, air brings the sacrificial materials to the rear part of the channel, which makes the transmittance of the rear part lower than that of front part of the channel. Additionally, ethanol would blur the surface of the channel. Although ethanol can remove most of the sacrificial materials, the transmittance of the chip is still low. Vegetable oil is the optimized remover due to the evidently higher transmittance. Furthermore, another build material (VisiJet^®^ Techplast material, gray, 3D Systems) was investigated and removed as a sacrificial material by ethanol and oil (Figures S7–S9). The results show that ethanol could blur the channel of the chip (Figures S7–S9).

In addition, processing by air has a greater variance than the other two methods and has the lowest transmittance among the three removers. From the results of processing by air, the transmittance of the inlet is more than twice that of the rear part and the outlet of the chip. This illustrates that air can be used as a remover in a short channel. Although processing by ethanol is a little better than processing by air, it is much lower than processing by vegetable oil. Therefore, the best remover among these three is vegetable oil. Air can be used when only a little sacrificial material remains.

### 3.4. Removing Inner Sacrificial Materials with Different Temperatures

To remove inner sacrificial materials, the sacrificial materials should be first melted. Melting temperatures should be studied. The basic material of the sacrificial materials in 3D-printed microfluidics is wax. The melting point of the sacrificial materials is around 60 °C. Herein, the chip and vegetable oil are heated to different temperatures (60 °C, 70 °C, and 80 °C) to remove the inner sacrificial materials.

**Figure 5:**
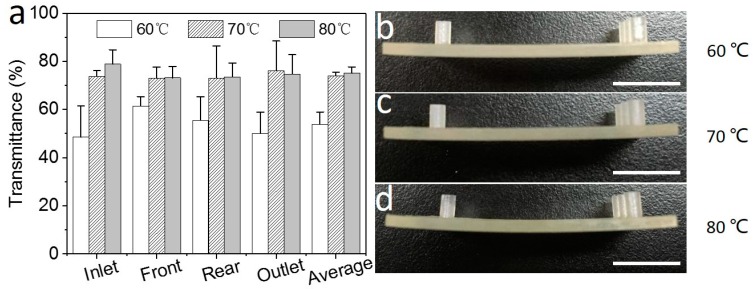
Removing inner sacrificial materials with different temperatures. (**a**) The microchannel transmittances of different parts in the chip and the average after processing with 60, 70, and 80 °C hot vegetable oil. (**b**–**d**) Chip deformation after processing at (**b**) 60 °C, (**c**) 70 °C, and (**d**) 80 °C. The scale bars are 1 cm.

After removing the inner sacrificial materials by heating the chip to 60 °C, the microscopic images of the specific four parts in the chip are acquired with the same conditions. Additionally, the grey scale values of the four parts are extracted from them (Figure S10). The transmittances of the four parts are 48.5% ± 13.0%, 61.5% ± 3.9%, 55.5% ± 9.8%, and 50.0% ± 8.8%, respectively (Figure S11). The average transmittance is 53.9% ± 5.1% (Figure 5a). Heating the chip to 70 °C, gray scale values of the four parts of the chip are presented (Figure S6). The transmittances of the four parts are 73.7% ± 2.5%, 73.0% ± 4.61%, 73.06% ± 13.28%, and 76.1% ± 12.36%, respectively (Figure S5). The average transmittance is 74.0% ± 1.3%. Heating the chip up to 80 °C, gray scale values are extracted as described above (Figure S12). The chip transmittances of the four parts are calculated (Figure S13). The chip transmittances of the four parts and the average transmittance are 78.3% ± 6.0%, 73.2% ± 4.8%, 73.6% ± 5.8%, 74.7% ± 8.1%, and 75.1% ± 2.2%, respectively.

The statistics results show that the transmittance increased with the increase of temperature when the temperature was below 70 °C. When the chip is heated to 60 °C, the transmittances of the four parts are obviously different. It indicates that the sacrificial materials are not melted completely and a higher temperature is needed. When the temperature increases to 70 °C, there is no obvious difference among the transmittances of different parts of the chips and the transmittance increased by 20.1% compared to 60 °C. Increasing the temperature to 80 °C, the transmittance only increases by 1.1% compared to 70 °C. Obviously, the increase rate decreases dramatically. This indicates that most sacrificial materials could be melted and removed at 70 °C. Moreover, the chip deformation, which could be caused by a high temperature, is also considered. When the chips are heated to 60 °C and 70 °C, the chip deformation is inconspicuous (Figure 5b,c). However, increasing the temperature to 80 °C, the chip is obviously bended (Figure 5d). Additionally, it may result in an unexpected change of the microchannel characteristics and disable the printed chips. Therefore, 70 °C is the optimized temperature when removing inner sacrificial materials in the channel.

### 3.5. Removing Inner Sacrificial Materials with Different Times

In this section, transmittances of the chips are measured with the extension of time (1, 3, 10, 20, 40, 120, 360, and 720 min) to find the cost-effective condition. For 1, 3, 10, 20, 40, 120, 360, and 720 min (Figures S14–S29), the transmittances are 37.5% ± 3.2%, 73.9% ± 3.2%, 73.7% ± 3.9%, 78.0% ± 4.7%, 78.9% ± 4.4%, 80.6% ± 7.8%, 88.8% ± 1.1%, and 93.8% ± 6.8%, respectively. The longer the removing time, the higher the transmittance of the chips. The transmittance changes quickly in the first 3 min (Figure 6). After 3 min, the transmittance rises more slowly.

There is almost no difference between the transmittance after 3 min and 10 min of removal. The transmittance of these two time points is nearly 74%. In the first 10 min, most sacrificial materials are melted and removed. The standard deviation of the chips after 10 min of removal is lower than that of the chips after 3 min of removal. A lower standard deviation means more stable results. In this respect, chips after 10 min of removal are more reasonable than chips after 3 min of removal. The transmittance of the chip is 93.8% after 720 min of removal. To observe the overall performance, images of the whole chips after different times of removal are obtained (Figure S30). Unlike chips after 1 min of removal, there is little difference that can be observed on the chips after 10, 40, and 720 min of removal. According to Sochol et al. [18], a dye-colored fluid is used to evaluate the channel performance between 10 and 720 min of removal. Herein, the fluid can flow through the channel and can be observed clearly (Figure S31). This illustrates that a transmittance of 73.7% does not affect the observation and fluid experiments. Chips after 10 min of removal can be used for microfluidic experiments.

Unlike researchers in the lab who are keen to find the best conditions and results, engineering techniques need to balance between costs and better results. Hence, the appropriate removing time is 10 min in this engineering technique designed for industrial applications.

### 3.6. Removing Sacrificial Materials with Different Cross-Sections

**Figure 7:**
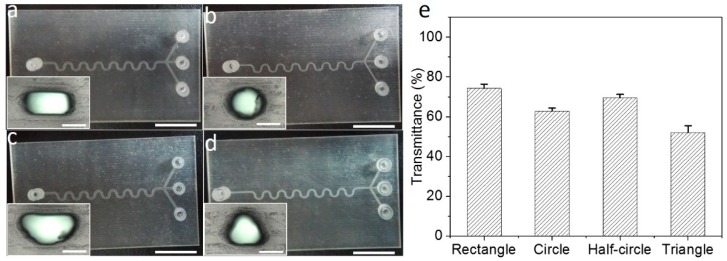
Removing inner sacrificial materials of 3D-printed microfluidic chips. Chips with a (**a**) rectangle, (**b**) circle, (**c**) half-circle, and (**d**) triangle cross-section, respectively. (**e**) The microchannel transmittances of different chips after processing with the optimized engineering technique. The scale bars of full images and enlarged images are 1 cm and 200 μm, respectively.

To confirm the general applicability of the obtained optimized conditions, microfluidic chips with different cross-sections, including a rectangle cross-section (Figure 7a), a circle cross-section (Figure 7b), a half-circle cross-section (Figure 7c), and a triangle cross-section (Figure 7d), are designed and 3D-printed. The width and height of the rectangle channel are 400 μm and 200 μm, respectively. The diameter of the circle channel is 267 μm. The diameter of the half-circle channel is 533 μm. The length of the equilateral triangle’s side is 533 μm. Based on these designs, the hydraulic diameter of each cross-section maintains the same value.

The optimized engineering technique is applied for removing sacrificial materials from each chip. The microchannel transmittances of the chips with different cross-sections are 74.3% ± 2.1% (rectangle), 62.8% ± 1.5% (circle), 69.5% ± 1.7% (half-circle), and 52.0% ± 3.5% (triangle), respectively (Figure 7e, Figures S32–S39). The microchannel transmittance of the chip with the rectangle cross-section is the highest among these four chips. The other two are the chips with the half-circle cross-section and the circle cross-section, and the lowest is the chip with the triangle cross-section. Meanwhile, the statistics results show that all the transmittances of the four chips still remain stable after 3 min of treatment (Figure S40). It indicates that the difference of transmittances is mainly due to the inherent optical properties (e.g., the way of refractions) of the microchannel with different cross-sections. Therefore, this engineering technique can be generally applied for post-processing of 3D-printed microfluidic chips with different cross-sections. In other words, the robustness and general applicability of the proposed engineering technique, including the inner/outer sacrificial materials removing approaches and their respective quantification methods, are confirmed.

## 4. Conclusions

In summary, this work presents engineering techniques for removing outer and inner sacrificial materials in 3D-printed microfluidic chips. For outer sacrificial materials removal, mass reduction (*R*) is proposed for quantification of the different removing approaches. Due to the highest mass reduction and lowest influence on the chip surface, it is found that the best method for outer sacrificial materials removing is a hot bath in vegetable oil. For inner sacrificial materials removing, the transmittance (*T*) of the microchannel is calculated as the quantification method. Based on this quantification method, vegetable oil is found to be the best remover for inner sacrificial material. Under a higher temperature, a higher transmittance of the microchannel could be reached, but a larger deformation of the chip is also observed. Thus, 70 °C is selected as the optimized removing temperature. Tested with a series of removing times, 10 min is proved to be a balance point between cost and chip performance. It is suggested that 10 min could be the appropriate removing time for this engineering technique when used in industry. Finally, this engineering technique with optimized conditions is applied in serval different cross-section chips to confirm its general applicability. It is indicated that this engineering technique has the potential to become a standard process for 3D-printed microfluidic chip fabrication, especially in industrial applications.

## Figures and Tables

**Figure 6:**
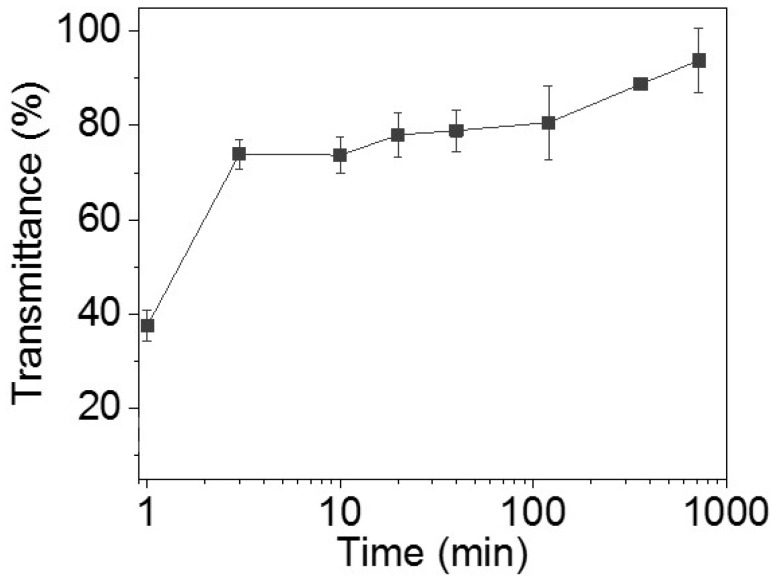
Microchannel transmittances of chips under optimized conditions as time goes on (1, 3, 10, 20, 40, 120, 360, and 720 min).

## Supplementary Materials

The following are available online at http://www.mdpi.com/2072-666X/9/7/327/s1, Figure S1: Gray scale value of chip images after removing sacrificial material by air; Figure S2: Transmittance of the chip after removing sacrificial material by air; Figure S3: Gray scale value of chip images after removing sacrificial material by ethanol; Figure S4: Transmittance of the chip after removing sacrificial material by ethanol; Figure S5: Gray scale value of chip images after removing sacrificial material by vegetable oil; Figure S6: Transmittance of the chip after removing sacrificial material by vegetable oil; Figure S7: Removing sacrificial materials by ethanol and oil in a 3D-printed microfluidic chip with gray build materials (VisiJet^®^ Techplast); Figure S8: Gray scale value of chip (VisiJet^®^ Techplast material, gray) images after removing sacrificial material by oil; Figure S9: Gray scale value of chip (VisiJet^®^ Techplast material, gray) images after removing sacrificial material by ethanol; Figure S10: Gray scale value of chip images after removing sacrificial material at 60 °C; Figure S11: Transmittance of the chip after removing sacrificial material at 60 °C; Figure S12: Gray scale value of chip images after removing sacrificial material at 80 °C; Figure S13: Transmittance of the chip after removing sacrificial material at 80 °C; Figure S14: Gray scale value of chip images after 1 min removing; Figure S15: Transmittance of the chip after 1 min removing; Figure S16: Gray scale value of chip images after 3 min removing; Figure S17: Transmittance of the chip after 3 min removing; Figure S18: Gray scale value of chip images after 10 min removing; Figure S19: Transmittance of the chip after 10 min removing; Figure S20: Gray scale value of chip images after 20 min removing; Figure S21: Transmittance of the chip after 20 min removing; Figure S22: Gray scale value of chip images after 40 min removing; Figure S23: Transmittance of the chip after 40 min removing; Figure S24: Gray scale value of chip images after 120 min removing; Figure S25: Transmittance of the chip after 120 min removing; Figure S26: Gray scale value of chip images after 360 min removing; Figure S27: Transmittance of the chip after 360 min removing; Figure S28: Gray scale value of chip images after 720 min removing; Figure S29: Transmittance of the chip after 720 min removing; Figure S30: Chip images after different times of removal; Figure S31: Bright field and fluorescent chip images after 10 and 720 min removing; Figure S32: Gray scale value of a rectangle cross-section chip after 10 min removing; Figure S33: Transmittance of the rectangle cross-section chip after 10 min removing; Figure S34: Gray scale value of a circle cross-section chip after 10 min removing; Figure S35: Transmittance of the circle cross section chip after 10 min removing; Figure S36: Gray scale value of a half-circle cross section chip after 10 min removing; Figure S37: Transmittance of the half-circle cross-section chip after 10 min removing; Figure S38: Gray scale value of a triangle cross-section chip after 10 min removing; Figure S39: Transmittance of the triangle cross-section chip after 10 min removing; Figure S40: Transmittance results of four different cross-sections at 0, 1, 3, 5, and 10 min after removing inner sacrificial materials at 70 °C using vegetable oil.
